# Widespread synaptic density loss in schizophrenia follows molecular and network architecture

**DOI:** 10.1038/s41380-026-03717-x

**Published:** 2026-06-25

**Authors:** Sidhant Chopra, Patrick D. Worhunsky, Mika Naganawa, Xi-Han Zhang, Ashlea Segal, Loïc Labache, Edwina Orchard, Vanessa Cropley, Stephen Wood, Gustavo A. Angarita, Kelly Cosgrove, David Matuskey, Nabeel B. Nabulsi, Yiyun Huang, Richard E. Carson, Irina Esterlis, Patrick D. Skosnik, Deepak C. D’Souza, Avram J. Holmes, Rajiv Radhakrishnan

**Affiliations:** 1 Department of Psychology, Yale University, New Haven, CT USA; 2 Orygen, The National Centre of Excellence in Youth Mental Health, Parkville, Melbourne, VIC Australia; 3 Centre for Youth Mental Health, The University of Melbourne, Melbourne, VIC Australia; 4 Department of Psychiatry, Yale University, New Haven, CT USA; 5 Department of Radiology and Biomedical Imaging, Yale University, New Haven, CT USA; 6 Department of Neuroscience, Yale University, New Haven, CT USA; 7 Department of Psychiatry, Brain Health Institute, Rutgers University, Piscataway, NJ USA; 8 Ann S. Bowers Women’s Brain Health Initiative, University of California Santa Barbara, Santa Barbara, CA USA; 9 School of Psychology, University of Birmingham, Birmingham, UK

**Keywords:** Neuroscience, Schizophrenia

## Abstract

Converging neuroimaging, genetic, and post-mortem evidence highlights the fundamental role of synaptic density reductions in schizophrenia pathogenesis. However, the brain-wide spatial pattern of these alterations and the mechanisms underlying this patterning remain to be established. Here, using [^11^C]UCB-J radiotracer positron emission tomography (PET) imaging in individuals with schizophrenia (*n* = 29) and healthy controls (*n* = 93), we find a prominent and widespread pattern of lower synaptic density (0.58 < Cohen’s *D* < 1.47; p_FWE_ < 0.05) in patients. The left hemisphere is substantially more impacted than the right (Cohen’s *D* = 1.14; *p* < 0.001), with frontal, temporal, cingulate, thalamic, striatal and hippocampal areas particularly affected. Synaptic density alterations were not spatially aligned with grey matter volume alterations indexed using anatomical Magnetic Resonance Imaging. Lower synaptic density in the left hemisphere is associated with higher normative concentrations of GABA_A/BZ_, 5HT_2A_, mGluR5 and 5HT_1B_ (*r*_*cca*_ = 0.68; *p* = 0.022). Simulation-based network diffusion models identified regions that may represent the initial sources of pathology, nominating left inferior frontal areas (*p*_*FWE*_ < 0.05) as potential foci from which synaptic pathology initiates and then propagates to structurally connected and molecularly similar areas. Overall, our findings provide in vivo evidence for widespread synaptic density deficits in schizophrenia that are left-lateralised, independent of grey matter volume alterations, aligned to specific neurochemical systems, and suggest that such synaptic pathology may propagate in a pattern consistent with axonal networks.

## Introduction

Post-mortem, genetic and neuroimaging studies have converged to implicate loss of synapses as a fundamental pathophysiological process in schizophrenia [1–4]. The advent of positron emission tomography (PET) radioligands with high specificity for synaptic vesicle proteins [5] has made it possible to examine presynaptic synaptic terminal density in vivo [6], with preliminary studies in schizophrenia [7–9] demonstrating reductions in specific cortical and subcortical areas. However, the brain-wide spatial patterning of synaptic pathology, and the mechanisms shaping this pattern, remain poorly understood.

Research in neurological conditions shows that the brains vulnerability to pathology is not spatially random but instead reflects intrinsic properties of large-scale systems [10], cellular composition [11], and receptor distributions [12]. Similar principles may apply in schizophrenia, where grey matter morphological deficits preferentially impact higher-order association networks and align with normative receptor expression profiles. Hemispheric asymmetry may represent another organising feature of this spatial patterning, with repeated evidence of a preferential pathology within the left hemisphere across micro (molecular [13, 14], cellular [15]), and macro (in vivo estimates of brain anatomy and function [16, 17]) scales. Moreover, several lines of evidence suggest that brain pathology in schizophrenia is constrained by macroscale axonal networks. For instance, grey matter reductions in patients correlate with the microstructure of adjacent white matter [18], across spatially distributed regions [19, 20], with normative connectome organisation [21, 22], and with reductions in structurally connected and functionally coupled regions [23–25]. Moreover, multiple longitudinal and modelling-based studies have now demonstrated that pathology may spread from an initial site to axonally connected territories [25–28]. However, widely used MRI-based morphometric measures predominantly index a composite of tissue properties such as intracortical myelin, neurite packing, glial density, iron and water content and vasculature, rather than synaptic terminals. Thus, direct in vivo measures are required to test whether the systems-level features confer vulnerability and shape the spatial organisation of synaptic pathology in schizophrenia.

Here, we address this gap by mapping the in vivo brain-wide spatial pattern of synaptic density alterations using [^11^C]UCB-J PET in a large single-site cohort of 122 individuals, including 29 with schizophrenia. Our primary hypothesis was that, relative to healthy controls, individuals with schizophrenia would show widespread reductions in synaptic density, particularly in left-hemisphere and higher-order association areas. We further hypothesised that the spatial patterns of lower synaptic density would overlap with MRI-derived grey matter volume alterations, covary with normative receptor/transporter architecture, and conform to a network diffusion process across spatially distributed parcels constrained by their associated structural connectivity. Our findings demonstrate a prominent, widespread pattern of reduced presynaptic terminal density in schizophrenia, with stronger effects in the left hemisphere and in frontal, temporal, cingulate, thalamic, striatal, and hippocampal regions. These reductions were spatially independent of grey matter volume alterations measured with structural MRI and closely aligned with normative distributions of specific neurotransmitter and transporter systems. Network diffusion models further nominated left prefrontal regions as potential sources of pathology, from which synaptic deficits may propagate to axonally connected and molecularly similar territories. Together, these findings provide the first in vivo evidence that presynaptic terminal deficits in schizophrenia are widespread, left-lateralized, and shaped by both molecular architecture and macroscale network organisation.

## Methods

### Sample characteristics

A total of 122 individuals, including 29 individuals diagnosed with schizophrenia were included in this study. Individuals with schizophrenia were recruited via clinician referrals, paper, and web advertisements. The diagnosis of schizophrenia was based on DSM-5 criteria for schizophrenia, which was confirmed by a comprehensive screening assessment that included a Structured Clinical Interview for DSM-5 Axis I Disorders, Clinician Version (SCID-CV), assessment by a research psychiatrist and past medical records. The majority of individuals with schizophrenia were recruited using criteria that excluded based on the following: regular exposure to drugs of abuse (except nicotine and caffeine) within the past 3 months based on history or positive urine drug screen, lifetime substance use disorder (except for nicotine and caffeine), weekly alcohol consumption exceeding National Institute on Alcohol Abuse and Alcoholism (NIAAA) guidelines (4 or more drinks on any single day and/or 21 or more drinks per week for males), history of significant head injury resulting in unconsciousness, unstable medical condition, significant prior exposure to radiation, metal in the body which would exclude MRI scan or claustrophobia. A subset of individuals with schizophrenia were recruited using a less restrictive criteria, where substance use was not an exclusion. We repeated our primary analyses after excluding subjects with a history of, or current, substance abuse (*n* = 7; SFig. 1). Data from 13 of the included individuals with schizophrenia have been part of a previously published study [7]. The healthy control subjects were included from studies performed at the Yale PET Centre using the [^11^C]UCB-J PET to investigate brain synaptic density across a variety of neuropsychiatric conditions [29]. Healthy control individuals were excluded for a current and/or lifetime diagnosis of a psychiatric disorder, current or past serious medical or neurological illness (including a history of head injury with loss of consciousness), metal in body which would result in MRI contraindication, or a history of substance abuse or dependence (regular exposure to nicotine or caffeine was not an exclusion; however one study excluded individuals who met criteria of a nicotine substance use disorder [*n* = 13] [30]). Sample size was informed by prior [^11^C]UCB-J schizophrenia studies which estimated that 9 participants per group were sufficient to detect hippocampal group differences under a one-tailed comparison at *α* = 0.05 and 80% power [9], while frontal and anterior cingulate cortex effect sizes reported in an independent [^11^C]UCB-J schizophrenia study correspond to an estimated sample size of approximately 16–21 participants per group [8]. Because ROI-based power estimates do not directly generalise to characterising brain-wide spatial patterns at the voxel level, which were the primary focus of the present study, these calculations were treated as lower-bound benchmarks. Demographic and sample characteristics are provided in Table 1. All study protocols were approved by the Yale University Human Investigation Committee and Radiation Safety Committee and all methods were performed in accordance with the relevant guidelines and regulations. All participants provided written informed consent prior to participation.

**Table 1:** Sample Demographics.

	Schizophrenia (*N* = 29)	Control (*N* = 93)	*p*-value
Age, mean years (SD)	43.21 (14.66)	45.40 (16.83)	0.501
Females, N (%)	4 (13.8%)	37 (39.8%)	0.018
Mass dose, mean in ug/kg (SD)	0.02 (0.02)	0.02 (0.02)	0.647
Activity at time of injection, mean MBq (SD)	235.53 (201.00)	194.61 (125.28)	0.332
T1w Image Quality Rating^a^ (SD)	1.89 (0.20)	1.87 (0.13)	0.683
Cum. Antipsychotic exposure, mean CPZ eq. (SD)^b^	2693.34 (2738.91)	–	–
Daily Antipsychotic dose, mean CPZ eq. (SD)	443 (346.90)	–	–
Education, Years (SD)*	13.03 (1.86)	–	–
PANSS Total, mean (SD)	68.39 (13.51)	–	–
PANSS Positive, mean (SD)	17.46 (5.26)	–	–
PANSS Negative, mean (SD)	16.64 (5.06)	–	–
PANSS General, mean (SD)	33.93 (6.88)	–	–
Age of illness onset, mean years (SD)	26.74 (11.33)	–	–
Duration of illness, mean years (SD)	15.19 (12.34)	–	–
Acutely psychotic or moderate illness, N (%)^c^	7 (24%)	–	–
Comorbid affective disorder, N (%)	12 (41.4%)	–	–
Handedness, N right (%)*	27 (93.1%)	–	–

^a^Derived using CAT12.

^b^CPZ eqs. = Cumulative (Cum.) lifetime exposure to antipsychotics as chlorpromazine (CPZ) equivalents (eq.). Medication type at time of imaging provided in SFig. 4.

^c^Defined by PANSS > = 75. Listed *p*-values correspond to two-sample t-tests, except for sex differences, where they correspond to a *Χ*^2^ proportion test. *PANSS* positive and negative syndrome scale.

*Handedness and Education were not consistently available for the healthy control cohort.

### PET and MR imaging acquisition and processing

[^11^C]UCB-J was synthesised using established methods [31]. All [^11^C]UCB-J PET measurements were conducted on the HRRT (Siemens Medical Solutions), which acquires 207 slices (1.2-mm slice separation) with a reconstructed image resolution of ~3 mm. Before every [^11^C]UCB-J injection, a transmission scan was performed for attenuation correction. Dynamic PET data (frames: 6 × 0.5 min, 3 × 1 min, 2 × 2 min, and 16 × 5 min) were acquired and reconstructed using the MOLAR algorithm [32]. Event-by-event motion correction was performed using Polaris Vicra optical tracking (NDI Systems, Waterloo, Canada) with reflectors mounted on the subject’s head via a swim cap [33]. Partial volume correction (PVC) was performed using the Müller-Gärtner method [34], which estimates the grey matter PET signal by correcting for spill-out from grey matter and spill-in from white matter and CSF using MRI-derived tissue maps and the PET point spread function. GM, WM, and CSF probability maps were generated from the T1-weighted MRI using CAT12 and, consistent with the original three-compartment Müller-Gärtner framework, converted to hard labels by assigning each voxel to the tissue class with the highest probability, such that no additional GM probability threshold was applied. The assumed PET spatial resolution for Müller-Gärtner correction was 3 mm FWHM. Then, the simplified reference tissue model 2 (SRTM2) [35, 36] with the centrum semiovale as the reference region, was applied using 60 mins of dynamic data to generate parametric images of binding potential (*BP*_ND_) [6, 7, 37, 38]. Mean voxel-level *BP*_ND_ maps for patients and controls are provided in SFig. 2.

A high-resolution T1-weighted structural MPRAGE scan (TR/ TE = 2530/3.34, flip angle = 7°, in-plane resolution = 0.98 × 0.98 mm, matrix size = 256 × 256, slice thickness = 1 mm, slices = 176) was acquired on a 3 T MAGNETOM Prisma scanner (Siemens, Erlangen, Germany). Registration of parametric PET images to Montreal Neurological Institute (MNI152) standard space was performed using SPM12 (Wellcome Trust Centre for Neuroimaging, London, UK) [39]. For each subject, PET images were motion-corrected by registering each frame to a summed image (0–10 min post-injection). A linear registration aligned the summed PET image to the T1-weighted MR image, followed by a nonlinear registration to the MNI template. The combined transformations were then applied to the parametric PET image. The standardised parametric PET images were smoothed with a 4 mm full-width half-maximum Gaussian kernel, then masked using an enhanced grey-matter tissue prior map thresholded at 0.25 [40].

### Mapping brain-wide synaptic density alterations

**Fig. 1:**
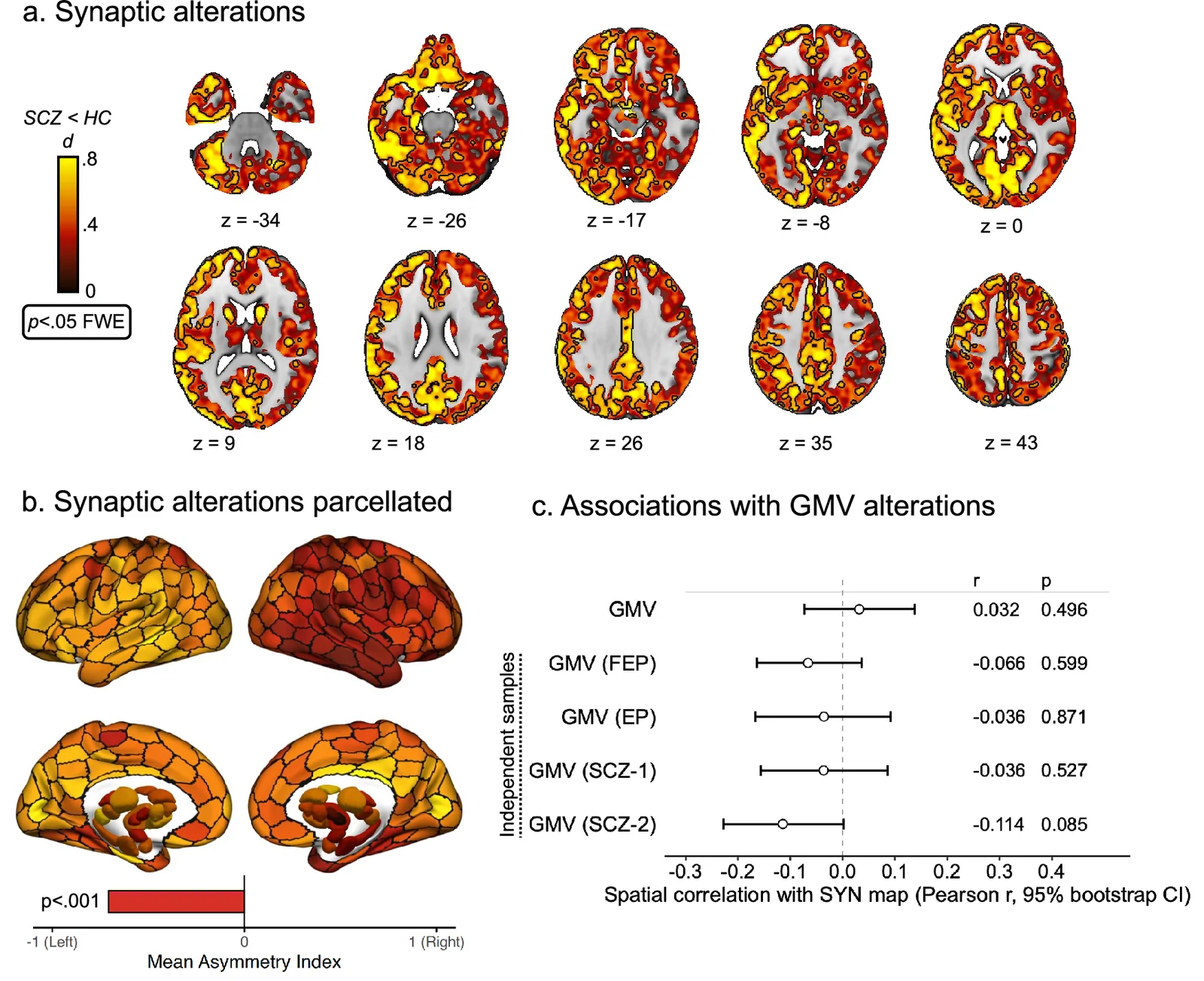
Widespread and left-lateralised lower synaptic density in schizophrenia. **a** Voxel-level effect-size map of difference in binding potential (non-displaceable) between individuals with schizophrenia (SCZ) and healthy controls (HC), adjusting for age and sex. Test statistics (t-values) were converted to standardized effect-sizes (Cohen’s *D*), with the black outline indicated voxel-level family-wise error corrected (FWE) significance at *p* < 0.05. **b** Effect-size map parcellated using the 300 homotopic cortical regions [42] and subcortical Tian 32 region [43] atlases, with the mean effect-size value of voxels assigned to each region. Bottom bar displays mean asymmetry index across all regions, with the *p*-value representing overall significant leftward laterality in synaptic loss. **c** Forest plot of spatial correlations (Pearson’s r) between parcellated synaptic density alterations (SYN) and grey matter volume (GMV) within the current sample (top row), as well as four independent samples including antipsychotic-naive first-episode psychosis (FEP), early psychosis (EP) and two established schizophrenia (SCZ) cohorts. No association between synaptic density alterations and grey matter alterations were found. Correlations between GMV maps between the current and independent samples are provided in SFig. 3. *P*-values were derived using non-parametric spatial autocorrelation preserving null models.

General linear models examining lower *BP*_ND_ in individuals with schizophrenia compared to controls, adjusting for age and sex, were fit at each grey matter voxel. Non-parametric voxel-level family-wise error (FWE) correction was implemented using CAT12 [41], using 5000 permutations, with significance assessed at p_FWE_ < 0.05 (Fig. 1a).

To examine hemispheric asymmetry in brain-wide synaptic pathology, t-statistic maps, which indexed differences in synaptic density between patients and controls, were then parcellated using a previously validated functional homotopic atlas into 300 discrete cortical [42] and 32 subcortical [43] areas of approximately equal size. Hemispheric asymmetry was quantified by computing right-left differences in the test-statistic across each homotopic parcel (negative values indicating leftward asymmetry). A one-sample t-test was used to test for systematic asymmetry. To generate a non-parametric null distribution, left and right values were randomly swapped within each homotopic pair (10,000 permutations), and significance was assessed as the proportion of permuted mean differences exceeding the observed mean difference.

### Association between grey-matter volume and synaptic density alterations

Voxel-based morphometry (VBM), implemented in the CAT12 toolbox [41], was used to index brain-wide grey matter volume alteration between individuals with schizophrenia and controls within the current sample, as well as four previously published independent samples [25] which included individuals with medication-naive first-episode psychosis [44] (N_case/control_ = 59/27), early psychosis [45] (N_case/control_ = 121/57), and two cohorts with established schizophrenia [46, 47] (N_case/control_ = 66/72; N_case/control_ = 70/62). The VBM procedure used for each sample has been previously described in detail [25]. The resulting brain-wide voxel-level t-statistic map from each study, which indexed differences in grey matter volume between patients and controls, was then parcellated using the same 332 region atlas described above. The volume alteration of a region was estimated as the mean test-statistic (comparing patients and controls) of all voxels corresponding to that region. The same parcellation procedure was followed for the synaptic density alteration map from the current sample (Fig. 1b), allowing for direct comparison between the synaptic and grey-matter volume alteration maps. Pearson correlations (r) were used to examine spatial associations between alterations in synaptic density and grey matter within the current sample and four independent cohorts (Fig. 1c). Statistical significance of associations between spatial maps was assessed using a benchmark null model that preserved spatial autocorrelation, allowing us to evaluate whether the observed findings were specific to the empirically observed alteration maps or were a generic property of the intrinsic spatial structure of these maps. The procedure used a spin-test to rotate cortical region-level t-values from the synaptic density alteration map 10,000 times [48, 49]. For brain-wide analyses, the rotation was applied to one hemisphere and then mirrored for the other hemisphere. For hemisphere-specific analyses, the rotations were computed independently for each hemisphere. Sub-cortical t-values were randomly permuted without replacement. All *p*-values were quantified as the fraction of null correlation values exceeding the observed correlation, considering both positive and negative tails of the null distribution (two-tailed). Statistical significance was assessed at *p* < 0.05 and inference was FWE corrected using Bonferroni correction (Fig. 1c).

### Quantifying network, neurochemical and cellular enrichment of synaptic density alterations

#### Network-level enrichment

**Fig. 2:**
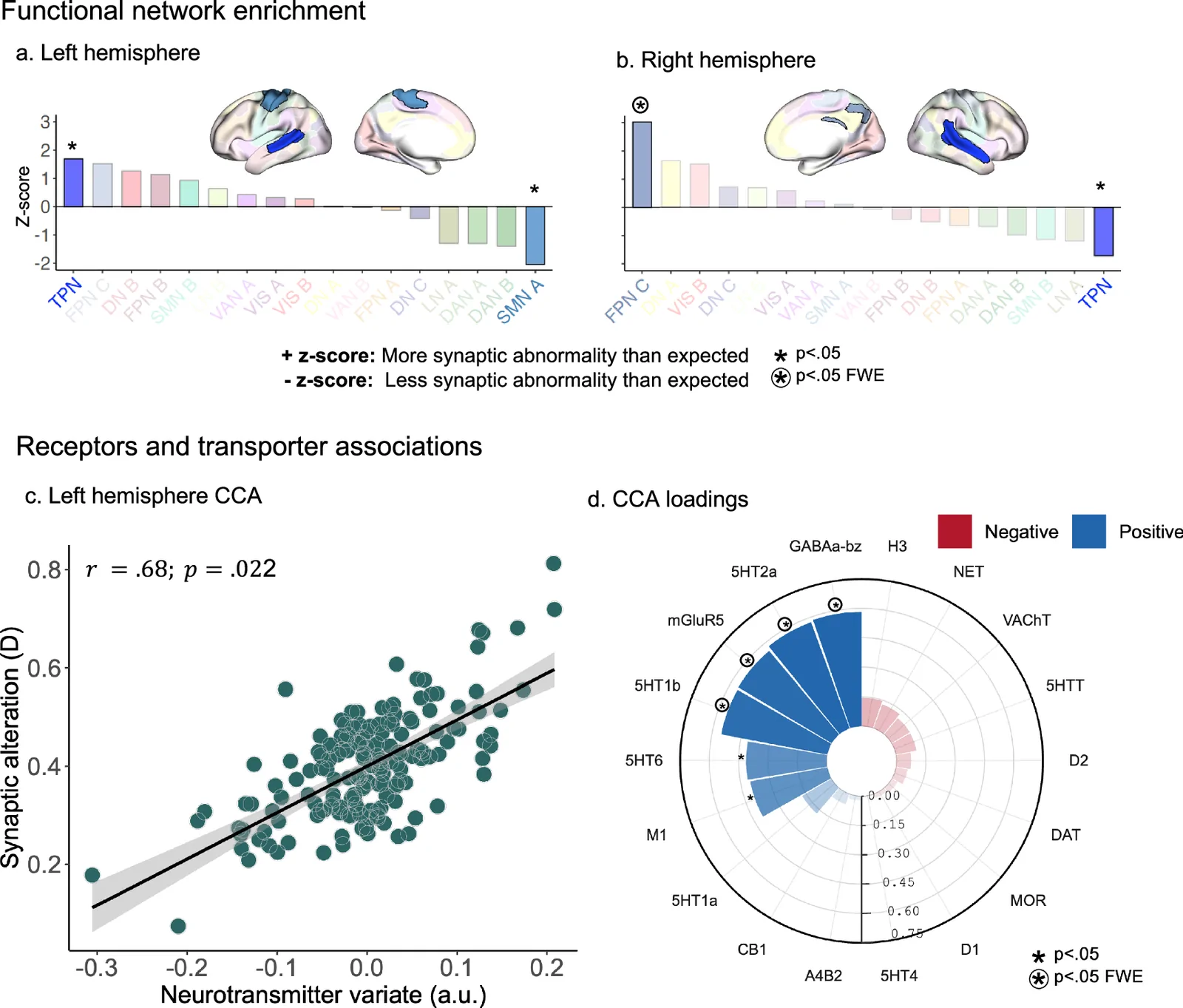
Network and neurochemical correlates of synaptic pathology in schizophrenia. **a,** **b** Enrichment of synaptic pathology within left (**a**) and right (**b**) hemisphere functional networks, defined using a 17-network cortical atlas [50, 51]. Bars plots show each network’s enrichment effect size as corresponding z-scores relative to the null distribution generated by the spatial permuting procedure. A positive z-score indicates greater synaptic loss than expected, whereas a negative z-score indicates less synaptic loss than expected. Asterisks (*) indicate statistical significance (*p* < 0.05), with a circle indicating survival against family-wise error (FWE) correction. **c** Significant canonical correlation between regional synaptic density deficits and normative receptor/transporter density in the left hemisphere. This panel shows the overall association between the synaptic deficit map and the canonical neurochemical variate, which is a weighted linear combination of all receptor/transporter maps. **d** Canonical loadings from the same significant canonical correlation analysis shown in panel c. These loadings indicate which individual receptor/transporter maps contributed most strongly to the canonical neurochemical variate. Positive loadings indicate that higher normative receptor/transporter density is associated with greater synaptic loss. Asterisks (*) indicate statistical significance using bootstrapping (*p* < 0.05), with a circle indicating survival of FWE correction. TPN temporoparietal network, DN default network, FPN frontoparietal network (sometimes referred to as cognitive control network), LN limbic network, VAN salience/ventral attention network, DAN dorsal attention network, SMN somatomotor network, VIS visual network.

To test whether synaptic pathology was preferentially enriched within specific functional or cytoarchitectonic systems, we used two brain classification schemes applied separately to each hemisphere: intrinsic functional networks (17-network Yeo atlas [50, 51]) and cytoarchitectonic types of laminar differentiation (von Economo–Koskinas atlas [48, 52, 53]). Synaptic pathology was estimated by averaging the t-statistic across regions belonging to each functional network or cytoarchitectonic subdivision (Fig. 2a, b; SFig. 5A-B). Statistical significance of observed systems-level synaptic pathology was compared to mean values derived using the spin-test with 10,000 permutations, as previously described. Statistical inference was assessed at *p* < 0.05 (two-tailed) and FWE corrected using Bonferroni correction across 17 functional networks and 7 cytoarchitectonic networks. To quantify the effect size for each network, we computed z-scores by comparing the observed test statistic to the null distribution [23].

#### Neurochemical and cellular associations

To examine whether synaptic density alterations were preferentially aligned with normative neurochemical or cellular densities, we used two sources of data. For regional normative neurochemical densities, we used 18 different group-consensus PET binding maps from healthy populations collated by the *neuromaps* toolbox [54]. Following previous work, for tracers that had multiple samples, a sample-size weighted average map was computed [55]. For cases where two different tracers targeted the same receptor/transporter, the one with the larger sample size was retained. Further details regarding each PET map are provided elsewhere [54]. Each PET map was masked and parcellated into the same 332 region atlas as the synaptic density alteration maps. Regional transcriptomic cell-type concentrations were deconvolved from whole brain bulk tissue transcriptions provided by the Allens Human Brain Atlas (AHBA) [56], with 24 different cell-types being imputed using recently published single-nucleus RNA-sequencing (snRNA-seq) data [57] (see Zhang et al [58] for detailed overview). Briefly, the transcription signatures identifying each class of cell in the AHBA bulk samples were derived from cortical snRNA-seq data of eight cortical areas. This included 24 cellular classes with distinct laminar specialisation, developmental origin, morphology, spiking pattern, and broad projection targets [59]. These cells include 9 GABAergic inhibitory interneurons (PAX6, SNCG, VIP, LAMP5, LAMP5 LHX6, Chandelier, PVALB, SST CHODL, SST), 9 glutamatergic excitatory neurons (L2/3 IT, L4 IT, L5 IT, L6 IT, L5 ET, L5/6 NP, L6 CT, L6b, L6 IT Car3), and 6 non-neuronal cells (Astro, Endo, VLMC, Oligo, OPC, Micro/PVM). This procedure resulted in cell density estimates for each of the 24 cell-types at each cortical region using the same atlas as the synaptic density alteration maps. Due to data availability, non-cortical areas were excluded for analyses that used these cellular data.

Given that regional neurochemical and cellular profiles are multivariate, we examined their association with synaptic density alterations using robust non-parametric canonical correlation analysis [60]. Separate CCA models were computed for receptor/transporter densities and cell-type densities. In each model, CCA tested the association between the regional synaptic density alteration map and a canonical variate (r_cca_) representing a weighted linear combination of the input features, that is, the 18 PET receptor/transporter maps or 24 cell-type density maps. Statistical significance of the observed canonical correlation coefficient was assessed against null correlations derived using the spin-test with 10,000 permutations. Statistical inference was assessed at *p* < 0.05, two-tailed. For significant CCA models, canonical loadings were quantified by correlating each individual input map with its corresponding canonical variate, thereby identifying which features contributed most reliably to the multivariate association. To assess statistical significance of canonical loadings, we used robust bootstrapping to estimate the standard error for each input map (10,000 bootstraps), computed z-scores by dividing the correlation by the standard error and used these z-scores to compute two-tailed *p*-values [61]. Maps showing reliable loadings on the canonical variate were those that survived a Bonferroni FWE-correction across input features (p_FWE_ < 0.05; two-tailed; Fig. 2d).

### Network diffusion model

**Fig. 3:**
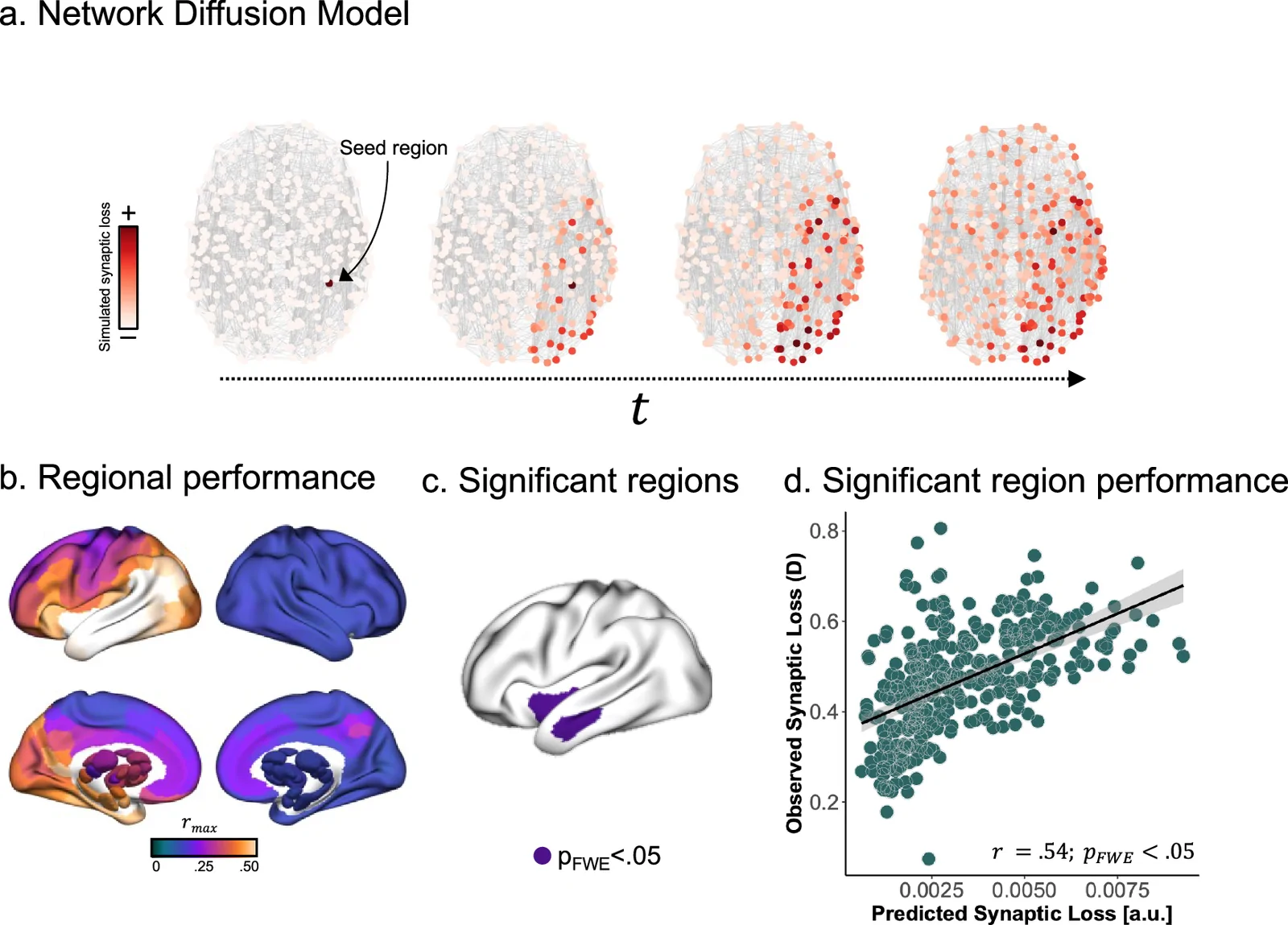
Network diffusion model of synaptic pathology. **a** To understand how global connectivity shape synaptic pathology, we simulated the spread of pathology using a network diffusion model. Using each of the 332 parcellated regions as a seed, we retained the maximum correlation between the simulated and observed synaptic abnormalities. We then compared maximum *r* values (r_max_) for each region to a distribution of maximum *r* values from two benchmark null models accounting for spatial autocorrelations in the synaptic pathology maps and basic topological properties of the connectivity matrix (see Methods). **b** Model performance (r_max_) for each region. **c** Seed regions (purple) with significantly (*p*_*FWE*_ < 0.05) greater r_max_ than both null models (**d**) Observed and network diffusion model predicted synaptic deficit, using the best performing seed (left frontal operculum/anterior insula).

We used a network diffusion model to test whether the observed pattern of lower synaptic density (Fig. 1b) is consistent with a process that propagates through the brain along structural connections, and to identify which regions may act as potential sources, or initiation sites, of this spread (Fig. 3) [62]. The model simulates pathology spreading across a weighted brain network, such that diffusion occurs preferentially between regions that are more strongly connected (Fig. 3a). The model was constrained by a weighted structural connectivity matrix as the base of diffusion modelling, which was derived using tractography applied to high-resolution diffusion-weighted imaging data from the Human Connectome Project [63] (details of the procedure provided in the Supplement).

Given that the simulated diffusion process can be started from any region in the brain, we repeatedly initialised the model using each of the 332 cortical [42] and subcortical [43] regions as the starting seed, and each time allowed the simulated process to spread throughout the brain to each other region. In this way, we were able to determine whether a diffusion process seeded from any of the regions resulted in a spatial distribution of synaptic density loss that matched the empirically observed patterns.

Consistent with prior work [25, 62, 64], model performance for each seed was quantified as the maximum Pearson correlation between the simulated diffusion pattern and the observed synaptic abnormality map across the simulation. In other words, for each seed region, we tracked how well the simulated pattern matched the observed pattern over time and retained the best match. The seed region itself was excluded from these correlations to avoid inflating model performance due to large synaptic alterations in the seed.

To assess statistical significance, we compared model performance of each seed regions predicted synaptic abnormality map against two benchmark null models: one based on spatially rotated surrogate maps (spin-test) and one based on rewired structural connectivity matrices. Seed regions were considered significant if they survived family-wise error correction (p_FWE_ < 0.05) in both null models. Full model equations, parameter settings, and null model procedures are provided in the Supplement.

## Results

### Widespread synaptic density abnormalities in schizophrenia

We find a prominent and widespread pattern of lower synaptic density in individuals with schizophrenia (Fig. 1a), compared to controls, with frontal, temporal, cingulate, thalamic, striatal, hippocampal and cerebellar areas surviving stringent voxel-level family-wise error (FWE) correction (0.58 < Cohens *D* < 1.47; *p*_*FWE*_ < 0.05, Fig. 1a). An analysis of hemispheric asymmetry indicated that the left hemisphere was substantially more impacted than the right hemisphere (*D* = 1.14, *p* < 0.001). We repeated these analyses after adjusting for antipsychotic medication exposure (SFig. 1A), after excluding individuals with a history of substance abuse (SFig. 1B), and after removing partial volume correction (SFig. 1C), finding a highly similar and significantly correlated pattern of lower synaptic density. Mean voxel-level *BP*_ND_ maps for patients and controls are provided in SFig. 2).

### Synaptic density abnormalities in schizophrenia do not overlap with grey matter abnormalities

The brain-wide spatial patterns of synaptic and volumetric alteration were not correlated within the current sample (*r* = 0.03; *p* = 0.496), nor across volume alteration maps from four independent samples (Fig. 1c). This was not because the current sample was neuroanatomically distinctive, since there were significant correlations between the volume alteration maps of the current sample and those from other datasets (0.33 < *r* < 0.41; *p*_*FWE*_ < 0.05;SFig. 3A), except for the first episode psychosis dataset (*r* = 0.−0.02; *p* = 0.81). This is consistent with a reduced magnitude of grey matter alterations reported in first episode psychosis, compared to chronic schizophrenia. These results remained consistent when examining each hemisphere independently (SFig. 3A), when deformation-based, rather than voxel-based, morphometry was used to measure grey matter volume differences between groups (SFig. 3B) and when partial volume correction was not implemented for the synaptic density maps (SFig. 3C). In an exploratory brain-wide association analysis, we examined whether voxel-wise synaptic density was associated with PANSS total score, PANSS positive score, and PANSS negative scores, adjusting for age and sex. No associations survived family-wise error correction, and the unthresholded effect size maps are provided in the Supplement (SFig. 7).

Overall, these results demonstrate prominent and widespread lowering of synaptic density in schizophrenia, which is left lateralized and spatially independent of commonly reported MRI-derived grey matter volume alterations across different stages of illness.

### Network & molecular enrichment of synaptic pathology

Brain abnormalities in psychiatric and neurological disorders preferentially affect specific systems such as macroscale functional networks, distinct cytoarchitectonic regions and neurotransmitter systems [11, 65, 66]. This spatial organisation of pathology suggests that the functional and molecular properties of distributed brain regions may confer differential vulnerability to synaptic pathology. Supporting this conjecture, in the right hemisphere, we find evidence for preferential accumulation of synaptic pathology within aspects of the functional frontoparietal/cognitive control network (FPN), in particular, dorsal precuneus and posterior cingulate areas (FPN C subdivision; *Z* = 3.02; *p* < 0.001; Fig. 2a). We also find preliminary evidence that while the temporoparietal network is preferentially impacted in the left hemisphere (*Z* = 1.70; *p* = 0.047; Fig. 2b), it is relatively spared in the right (Z = −1.72; *p* = 0.025; Fig. 2a), suggestive of functional network-level asymmetry in synaptic pathology. No significant enrichment was observed for any cytoarchitectonic types of laminar differentiation (SFig. 5).

We found a significant multivariate association between the spatial pattern of synaptic density alterations and normative receptor/transporter densities within the left hemisphere (*r*_*cca*_ = 0.68; *p* = 0.022; Fig. 2c). Specifically, the canonical correlation analysis tested the association between the regional synaptic density alteration map and a canonical neurochemical variate, defined as a weighted linear combination of the receptor/transporter maps. Figure 2c shows the strength of the overall multivariate association, but it does not by itself indicate which specific receptor/transporter maps contributed most strongly to that relationship. To address this, Fig. 2d displays the canonical loadings from the same significant analysis, showing the contribution (i.e., loading) of each individual receptor/transporter map to the canonical neurochemical variate. Regions with higher normative densities of GABA_A/BZ_, 5-HTT_2A_, mGluR5, and 5-HTT_1B_ showed positive loadings that reliably contributed to the canonical variate (*p*_*FWE*_ < 0.05. Fig. 2d), indicating that these maps were driving the association with greater synaptic loss. No significant neurochemical association was detected in the right hemisphere. No significant cell-type associations were detected in either hemisphere and the corresponding cell-type CCA null results are provided in the Supplement (SFig. 6).

### Synaptic alterations may be constrained by white-matter pathways

To understand the spatiotemporal dynamics of synaptic loss in schizophrenia and to identify regions from where pathology may initiate, we used a network diffusion model [62]. This model directly tests whether synaptic loss propagates through the brain in a way consistent with a process of diffusion between axonally connected regions (Fig. 3a). The performance (*r*_*max*_) for each brain region when used as a starting seed is shown in Fig. 3b. Two regions, both in the left hemisphere showed the best performance and survived FWE correction (*p*_*FWE*_ < 0.05) across both null models: frontal operculum/anterior insula (*r*_*max*_ = 0.54; Fig. 3c) and the middle temporal gyrus (*r*_*max*_ = 0.53 Fig. 3c). Observed and predicted synaptic pathology using the best performing seed region (left frontal operculum/anterior insula) are shown in Fig. 3d. These findings suggest that synaptic pathology may initiate in left hemisphere inferior frontal and middle temporal regions and spread to structurally connected regions.

Given the previously identified association between neurotransmitter systems and synaptic alterations (i.e., Fig. 2c-d), we examined an exploratory network diffusion model in which the interregional connectivity matrix was weighted by receptor/transporter similarity between regions (see Supplement for details). In this way, the model preferentially diffused pathology not only between regions that were structurally connected, but between those that also share a similar neurotransmitter composition. In this model, the left frontal operculum/anterior insula areas again showed the best performance survived FWE correction (*p*_*FWE*_ < 0.05) across both null models, suggesting that synaptic pathology may propagate between structurally connected and molecularly similar areas.

## Discussion

Consistent with our primary hypothesis, we discovered a brain-wide pattern of lower in vivo synaptic density in individuals with schizophrenia, compared to a large sample of healthy participants. Although this pattern was left lateralized, contrary to our hypothesis of spatial overlap, it was independent of commonly reported MRI-derived grey matter volume alterations across different stages of psychotic illness. Partially consistent with our hypothesis, functionally defined frontoparietal/control areas were enriched for synaptic pathology in the right hemisphere, whereas the spatial pattern of lower synaptic density in the left hemisphere is strongly coupled to normative neurochemical composition, with areas rich in GABA_A/BZ_, 5HT_2A_, mGluR5, and 5HT_1B_ receptors most strongly affected. Using simulation-based network diffusion models, left inferior frontal/anterior insula areas were identified as initial sources from which synaptic pathology initiates and spreads to axonally connected and neurochemically similar areas.

### Widespread lower synaptic density in schizophrenia

The quantification of synaptic density within the living brain used [^11^C]UCB-J, a PET tracer that targets the Synaptic Vesicle Protein 2A (SV2A) [6, 67] which is localised and monodispersed on vesicular membranes within all chemical pre-synaptic boutons [68–70]. This tracer has highly specific binding to SV2A [31], good test-retest reliability [6, 71], and displacement within grey matter using a drug with specific binding to SV2A [6]. In primates, regional SV2A levels are strongly correlated (r > 0.95) with synaptophysin [6], a validated marker of ex vivo presynaptic density. Moreover, [^11^C]UCB-J uptake recapitulates anatomically inferred patterns of synaptic density [6], has been shown to be sensitive to synaptic pathology across multiple neurodegenerative conditions [6, 72–74], and is specific to synaptic loss measured by electron microscopy, rather than reductions in protein levels [75]. Nonetheless, it is possible that the alterations detected here represent reduced SV2A or vesicular organelles in schizophrenia, in the absence of changes in terminal numbers. For instance, initial evidence suggested that a polymorphism in the SV2A gene may increase risk for schizophrenia, though this has not been replicated in recent large-scale genome-wide association studies [76]. Furthermore, while sustained vesicular depletion occurs as a secondary effect in neurological disorders initiated by illness-linked proteins [77–79], limited post-mortem evidence in schizophrenia actually suggests increased numbers and clustering of synaptic vesicles [80, 81]—findings that may result from ex vivo artifacts [82, 83]. Given the sensitivity of [^11^C]UCB-J to detecting synaptic terminal loss in other neurological conditions, and the strong converging genetic and post-mortem evidence demonstrating lower presynaptic terminals and dendritic spines in schizophrenia, it is likely that lower levels of [^11^C]UCB-J binding detected here in schizophrenia are a result of lower numbers of synaptic terminals.

We find that areas of grey matter in individuals with schizophrenia, especially thalamic, striatal, hippocampal, frontal, temporal, cingulate, cerebellar and occipital regions, show lower levels of synaptic density compared to controls, with medium to large effect sizes (0.58 < *D *< 1.47). These findings align with previous region-of-interest studies examining in vivo synaptic density in early psychosis and schizophrenia populations, which have reported lower tracer binding in frontal, anterior cingulate, hippocampal, temporal, occipital and striatal regions [7–9, 84, 85], using both measures of specific binding to SV2A, such as that used in the current study, and measures that additionally index non-specific binding. Extending this prior work, our results suggest that synaptic alterations are not only isolated to these regions but are far more widespread and left lateralised in chronic schizophrenia than previously reported. Our findings are also consistent with large-scale neuroanatomical studies in schizophrenia showing subtle left-lateralised cortical thinning driven by regions of the language network [16]. An important consideration is that the present sample was experiencing chronic illness, which limits inference regarding the temporal origins of the observed synaptic abnormalities. Accordingly, we cannot determine whether these findings reflect primary illness-related mechanisms, cumulative illness progression, long-term treatment exposure, or a combination of these factors, and future longitudinal studies in earlier-stage and antipsychotic-naïve samples will be needed to disentangle these possibilities.

Patients with schizophrenia often exhibit alterations in cortical thickness and grey matter volume [86–91], which can progressively worsen in some individuals [44, 92, 93]. Given the limited evidence from post-mortem studies for a reduction in the number of neurons, it has been theorised that the substrate underlying these MRI abnormalities are alterations in synaptic, dendritic, and axonal organisation [94]. However, there is limited direct evidence linking MRI signals to neurite changes in psychotic illnesses. Alternatively, MRI signal differences between patients and controls may reflect differences in properties related to myelination, soma, glia, vasculature, or extracellular space. The lack of neurobiological understanding behind MRI signal alterations in psychotic illness have led others to suggest that artefactual alterations between cases and controls in hydration, cholesterol levels, physical and mental activity, and stress [95–97] may be causes of the observed volumetric changes. Although we hypothesised partial overlap, the spatial pattern of synaptic loss was largely independent of MRI-derived grey matter volume alterations, both within the current sample and across MRI data from independent psychosis cohorts across different stages of illness. This suggests that a lowering of presynaptic terminals might not serve as the primary substrate underlying grey matter MRI abnormalities in psychotic illness. This finding is also consistent with prior work demonstrating that age-related loss of synaptic density has a different spatial pattern from age-related MRI-derived grey matter volume loss [29]. However, this does not preclude partial convergence in selected regions or systems. Synaptic density and grey matter volume likely index related but non-equivalent pathological processes, which may be shaped by common network constraints while remaining dissociable at the whole-brain level. Moreover, future work should examine the association with a broader range of MRI-derived grey matter metrics, such as cortical thickness and surface area.

### Local molecular vulnerability and global axonal connectivity constrain synaptic abnormalities

In many neurological illnesses, pathological processes selectively target or initiate from specific genetic, cellular [98, 99], neurochemical [100, 101], and functional systems [10, 102]. Consistent with our hypotheses, the spatial pattern of synaptic pathology was related to both normative receptor/transporter architecture and macroscale network organisation. Specifically, we find evidence for the preferential accumulation of synaptic pathology within functionally defined control systems, specifically dorsal precuneus and posterior cingulate areas. This is consistent with a large body of work demonstrating that higher-order transmodal association areas are a primary site of pathology in psychosis [103, 104]. Our findings also indicate a strong spatial correspondence between left-hemisphere synaptic pathology and normative receptor profiles. Specifically, regions typically rich in GABA_A/BZ_, 5HT_2A_, mGluR5 and 5HT_1B_, receptors, show vulnerability to synaptic pathology. Although these receptors are predominantly post-synaptic, their spatial association with lower presynaptic terminal density may reflect both bidirectional synaptic coupling and a shared molecular environment. Post-synaptic signalling can influence presynaptic function via retrograde mechanisms [17], including endocannabinoid signalling, particularly for mGluR5 and 5-HT_2_ receptor systems [105, 106]. More broadly, post-synaptic dysfunction can disrupt trans-synaptic adhesion and scaffolding processes that help stabilise synapses, biasing them toward weakening, pruning, and coupled pre- and post-synaptic loss [107, 108]. This suggests that lower synaptic density may preferentially arise in molecular environments that shape synapse stability and vulnerability across these transmitter systems. Indeed, all three neurotransmitter systems have been shown to be disrupted or track symptom severity in schizophrenia [109–111], suggesting that synapses associated with these systems are vulnerable to pathological processes. This is also consistent with genetic findings showing that enrichment of common variant associations is restricted to genes that are expressed in both excitatory and inhibitory neurons [112, 113] and in genes encoding proteins involved in general synaptic features. Notably, we did not find associations with dopamine receptors or transporter, despite finding lower synaptic density in striatal regions. This may be because our brain-wide spatial analysis is dominated by variation in cortical patterns that overlaps poorly with the predominantly striatal distribution of dopamine maps. Moreover, dopaminergic abnormalities in schizophrenia are thought to reflect presynaptic functional dysregulation rather than regional receptor/synapse loss. Similarly, we also did not observe a significant association with the VAChT map, despite increasing evidence implicating cholinergic dysfunction in psychosis [114], including lateralisation of the system. One possible explanation is that cholinergic alterations most relevant to psychosis may be better captured at the level of muscarinic receptor signalling, consistent with our observed implication of M1 receptors, rather than presynaptic cholinergic terminal density indexed by VAChT. In addition, our neurotransmitter maps were derived from normative healthy PET data and therefore may not capture disease-related or subtype-specific cholinergic reorganisation in schizophrenia.

We also did not find that densities of transcriptomically-defined cell types were associated with the spatial pattern of synaptic pathology. Many post-mortem and genetic studies have shown layer specific cell abnormalities, commonly in frontal supragranular cells [115, 116] and particularly in cortical layer three [117–119]. The current spatial resolution of PET does not allow for layer specific inference, therefore a spatial correspondence with layer specific cell types may become apparent with improving resolutions.

We used a network diffusion model to understand if local molecular compositionand brain-wide axonal connectivity constrain the spatiotemporal dynamics of synaptic loss. We find that left frontal operculum/anterior insula and middle temporal cortices are a putative initial source of synaptic density loss in schizophrenia. Neuroanatomical alterations in these regions are some of the earliest neural alterations reported in the initial stages of illness [120–122]. Our exploratory analysis which parametrized the network diffusion model using a neurotransmitter similarity matrix again nominated left frontal operculum/anterior insula areas as putative source regions. These candidate epicentres differ from those identified in our previous work where network diffusion models were applied to grey matter volume alterations, which implicated the anterior hippocampus [25]. This discrepancy is consistent with our current finding that the spatial patterns of grey matter and synaptic alterations do not covary. However, studies using event-based models such as subtype-and-stage inference models (SuStaIn), rather than network diffusion models, have consistently identified a subtype, which is the most commonly occurring, in which inferior fronto-insular regions are affected early [26, 123]. Therefore, it is possible that synaptic and grey matter pathology are shaped by common macroscale vulnerability constraints yet reflect non-equivalent biological processes that yield different whole-brain spatial patterns and putative epicentres. Differences between SuStaIn and network diffusion findings may also reflect model objective, as SuStaIn is able to capture subtype-specific trajectories, whereas work using network diffusion models has thus far not accounted for intersubject variability in brain trajectories.

Overall, our results are in line with a pathological process that preferentially propagates via axonal connections to molecularly similar areas. The precise mechanism driving this process remains unclear. Trans-neuronal spreading processes have often been seen in prion-like spread of misfolded proteins, but limited evidence exists for visible deposits of aggregated pathological proteins in psychotic illness (although see [124–126]). However, although speculative, subtle changes in protein homeostasis [127] may interact with regional neurotransmitter levels [128, 129] and spread to synaptically connected distal brain region [130, 131]. Alternatively, and given the commonly reported finding of functional brain alterations in psychotic disorders, dysfunction of one region may trigger abnormal functional activity in connected sites that, over time, may trigger synaptic changes as a result of aberrant neurotransmission or a loss of trophic support [132]. Indeed, trans-neuronal propagation of excitatory-inhibitory imbalance, with consequent excitotoxic stress, could plausibly contribute to the reduced synaptic density observed in schizophrenia [133].

### Limitations

Our analyses of synaptic density depended on group-level summary values and may not directly reflect synaptic differences for individual patients. While the extent of interpatient heterogeneity in synaptic pathology is currently unknown, subsequent work could use larger samples with synaptic density PET imaging to derive patient-specific measures, such as those obtained through normative modelling [134, 135]. Handedness data were unavailable for healthy controls; however, given evidence that structural hemispheric asymmetries occur through mechanisms dissociable from handedness [136–138], we do not consider this to have substantively influenced the observed laterality pattern. Future studies should examine relationships between lateralised synaptic density and cognitive or language function, given the left-predominant distribution of findings reported here. The cross-sectional nature of this study precludes inference about the temporal onset of synaptic pathology, and further longitudinal studies will be able to clarify whether these alterations occur during neurodevelopment, at transition to illness or at later stages. An important consideration here is that the cell and receptor density maps used in the current study were derived from healthy samples and therefore represent normative cytoarchitecture and chemoarchitecture. Future data aggregation efforts examining cell-type and receptor maps from patient populations may provide additional evidence regarding synaptic vulnerabilities in schizophrenia. Our approach to characterize structural connectivity is also limited by the accuracy of diffusion MRI [139]. While the processing procedures we applied enhanced the biological validity of our structural connectivity measures as much as possible [140], further developments in non-invasive connectivity mapping and tractography will be required to allow more precise mechanistic inferences about constraints imposed by axonal pathways. A further limitation is that arterial blood sampling was not available across the full sample, precluding estimation of total distribution volume (V_T_). We therefore quantified [^11^C]UCB-J using BP_ND_ derived from a validated reference-region approach, which has been widely used for this tracer and shows good agreement with simplified non-invasive quantification methods [6, 7, 37, 38]. While white matter should have negligible specific binding, displacement studies showed small changes in the centrum semiovale, and subtle differences in reference-region binding could lead to misestimation of group difference [141]. Finally, the schizophrenia sample was predominantly male, whereas the healthy control group was more balanced with respect to sex. Although sex was included as a covariate in all analyses, residual sex-related effects cannot be excluded, and future studies with more balanced samples should more directly examine sex differences in SV2A PET measures and their spatial patterning in schizophrenia.

Overall, our findings provide in vivo evidence for widespread presynaptic terminal deficits in schizophrenia that are left-lateralised, independent of grey matter alterations, aligned to specific neurochemical systems, and suggest that synaptic pathology may propagate in a way consistent with axonal networks.

## Supplementary information

SUPPLEMENTAL MATERIAL

## Data Availability

Data are available from the senior author upon reasonable request, subject to principal investigator approval and ethics committee restrictions.
